# ADVANCE CARE PLANNING IN A SUBSIDIZED HOUSING COMMUNITY: PRELIMINARY RESEARCH RESULTS FROM A PILOT INTERVENTION

**DOI:** 10.1093/geroni/igae098.4386

**Published:** 2024-12-31

**Authors:** Christine Kimpel, Lorely Chavez, Christian Ketel, Kate Clouse

**Affiliations:** Vanderbilt University, Nashville, Tennessee, United States; Vanderbilt University, Nashville, Tennessee, United States; Vanderbilt University, Nashville, Tennessee, United States; Vanderbilt University, Nashville, Tennessee, United States

## Abstract

Advance care planning (ACP) can improve end-of-life outcomes, but ACP remains low among adults with low SES, who likely have had multiple adverse life experiences. To reduce the stress of ACP, we are piloting a trauma-informed advance care planning (TI-ACP) intervention in a subsidized housing community. Following IRB approval, we purposively and snowball sampled 15 adults (aged 18+) in a 150+ unit subsidized housing community in Nashville, Tennessee (May 2024-present). Using a pre-post single-arm intervention design, we conducted visits to collect mixed methods data in the onsite Resiliency Hub, a resource center. The intervention included a researcher-led conversation using a discussion prompt checklist. The median age was 60 (IQR: 57-65), seven (46.7%) identified as Black or African American, and eleven (73.3%) identified as men (n=15). At 30-day follow-up (n=9), implementation acceptability, appropriateness, and feasibility score medians and ranges were 16 (16-18), 16 (15-17), and 16 (15-18), respectively. Implementation barriers included competing priorities, limited social support, skepticism about outsiders, ACP skepticism, and medical mistrust. Potential adaptations included explaining privacy procedures and including supplementary education for challenging medical concepts. Participants rated the innovation as acceptable, appropriate, and feasible and provided insight into potential barriers and adaptations. The conversation checklist and educational materials may be utilized across clinical and community-based contexts. A team-based approach may maximize the benefit of TI-ACP by holistically addressing complex social and health barriers for older adults with low SES. Future iterations will explore using community health workers as interventionists and a community advisory council to design training.

